# Disturbances in attachment: inhibited and disinhibited symptoms in foster children

**DOI:** 10.1186/1753-2000-8-21

**Published:** 2014-07-15

**Authors:** Caroline S Jonkman, Mirjam Oosterman, Carlo Schuengel, Eva A Bolle, Frits Boer, Ramon JL Lindauer

**Affiliations:** 1 Department of Child and Adolescents Psychiatry, Academic Medical Center, University of Amsterdam and with De Bascule, Academic Center for Child and Adolescents Psychiatry, Meibergdreef 5, Amsterdam 1105 AZ, The Netherlands; 2 Department of Clinical Child and Family Studies and the EMGO Institute for Health and Care Research, VU University Amsterdam, Amsterdam, The Netherlands

## Abstract

**Background:**

Previous DSM-versions recognized an inhibited and a disinhibited subtype of the Reactive Attachment Disorder (RAD). The current DSM-5 distinguishes two different disorders, instead of two subtypes of RAD. This study examined whether a split-up of the subtypes is valid.

**Method:**

In 126 foster children, attachment disorder symptoms were assessed with the Disturbances of Attachment Interview. Forms of pathogenic care were identified based on dossier analyses. Associations between symptoms of attachment disorder with internalizing and externalizing problems (Child Behavior Checklist and Teacher Report Form) were examined.

**Results:**

Omnibus tests showed no significant association between type of symptoms and type of pathogenic care. Exploratory analyses did reveal an univariate association between disinhibited symptoms and history of physical abuse. Disinhibited symptoms were associated with more internalizing and externalizing problems (*d’s* < 0.50).

**Conclusion:**

The distinction of inhibited and disinhibited subtypes of RAD seems valid regarding their emotional and behavioral correlations. Whereas inhibited symptoms lack a correlation, disinhibited symptoms seem to have an externalizing and internalizing correlation.

**Trial registration:**

NTR1747

## 

According to DSM IV (Diagnostic and Statistical Manual of Mental Disorders 4th edition – text revision [1] Reactive Attachment Disorder (RAD) describes the clinical condition wherein children, as a consequence of pathogenic care, fail to seek proximity with a preferred caregiver and are unable to form an attachment relation. DSM IV distinguishes two forms of RAD, the inhibited and disinhibited subtype. Previous DSM versions acknowledged either type of RAD as part of the same disorder. The current DSM 5 separates RAD into two different disorders instead. Inhibited behaviors are still considered symptoms of the Reactive Attachment Disorder, but disinhibited behaviors are now described as symptoms of Disinhibited Social Engagement Disorder. Evidence that led to this division of RAD was primarily derived from a unique sample of Romanian institutionalized children (The Bucharest Early Intervention Project [2]). Other than the outcome from that unique institutionalized sample, little evidence is available to support the division of the inhibited and disinhibited subtype of attachment disorders. For that reason, in a sample of maltreated foster children, we examined similarities and differences between inhibited and disinhibited symptoms with regard to specific experiences of pathogenic care as well as problem behavior.

The inhibited subtype identifies children who have no preferred caregiver, rarely seek comfort in times of stress, show a minimum of positive affection, and/or experience difficulties in the regulation of their emotions. Inhibited symptoms are reported in children that lack selective attachment and symptoms tend to represent disturbances in attachment [3,4]. It has been suggested that the inhibited subtype comprises internalizing behaviors, but the only study that reported correlations between inhibition and internalizing problems was based on a limited number of children [4]. Because inhibited symptoms are rarely reported in follow-up studies in post-institutionalized children, it has been suggested that the inhibited subtype is responsive to enhanced caregiving quality [4,5].

The disinhibited subtype identifies children that may overly engage in contact with relatively strange adults and rarely socially discriminate between the caregiver and unfamiliar adults. Disinhibited symptoms also exist in children with selective attachment, and they have been reported in children with insecure and even secure attachment behaviors [6,7]. Symptoms of the disinhibited subtype have been associated with externalizing behaviors [8,9] and seem less responsive to improved caregiving quality [10].

Although the evidence that supported two distinctive disorders was mainly based on data from institutionalized (and formally institutionalized) children, DSM acknowledges other forms of pathogenic care responsible for the development of RAD. To extend findings to children exposed to other forms of pathogenic or low quality care, our study examined the emotional and behavioral correlates of inhibited and disinhibited symptoms in maltreated foster children.

To investigate the differential effects of distinctive pathogenic caregiving conditions, associations of the inhibited and disinhibited subtype with specific conditions were investigated. Because lack of selective attachment has been suggested to underlie inhibited symptoms, we hypothesized inhibited symptoms to be associated with a caregiving history characterized by absence of a preferred caregiver or discontinuity in caregiving (i.e. multiple placement breakdowns and neglect). Disinhibited symptoms have been linked to multiple forms of pathogenic care, identifiable in children with and without selective attachment. More than the processes leading to inhibited or disinhibited symptoms, difference may be found in the recovery from either inhibited or disinhibited symptoms. Because evidence suggested responsiveness to improved care of the inhibited subtype, we expected symptoms of this type to be negatively associated with time in foster care. Because disinhibited symptoms seem less responsive than inhibited symptoms to the improvement of caregiving, no association was expected between disinhibited symptoms and time in foster care.

Next, the emotional and behavioral correlations of both types were determined. In line with previous studies, we expected inhibited symptoms to be associated with internalizing and not with externalizing behaviors, whereas disinhibited symptoms were expected to be associated with externalizing and not internalizing problems.

Although the DSM-5 has split the previously existing category of reactive attachment disorder, which included both the inhibited and the disinhibited symptoms, into two separate diagnoses, co-occurrence of inhibited and disinhibited symptoms has been reported [11-13]. The overlap, however, was ascribed to assessment and statistical methods [14]. However, a mixed type of inhibited and disinhibited symptoms was included in the Research Diagnostic Criteria – Preschool Age [15]. These issues raise the question whether symptoms of the Reactive Attachment Disorder and Disinhibited Social Engagement Disorder can co-occur. Our study intended to further explore the existence of a mixed type of inhibition and disinhibition in relation to low quality of care and internalizing and externalizing problem behaviors.

## Methods

### Participants

We included 126 children (*M*age = 60.40 months, *SD*age = 15.53, age range = 26–89) in kinship and non-kinship foster families. The sample consisted of 50% boys and 50% girls. Almost all children (96%) had experienced at least one breakdown of a foster care placement (Number of placements; *M* = 2.48, *SD* = 1.50, range = 0–8). Children were between 0 and 78 months old when they were removed from their biological parents (*M* = 23.57, *SD* = 21.80). At the time of assessment children were between 2 and 76 months in the current foster family (*M* = 21.37*, SD* = 20.48). Children were recruited for either one of two studies. The first project studied attachment in 61 children in regular foster care (RFC; *M*age = 56.46 months, *SD*age = 16.48, age range = 26–88). Data from this study have been published before [16,17]. The second project studied attachment in 65 children in treatment foster care (TFC; *M*age = 64.11 months, *SD*age = 13.70, age range = 32–89). Contrary to foster children in RFC, foster children and parents in TFC receive intensive treatment (e.g. behavioral interventions, trauma therapy).

### Procedure

The study within the regular foster care project (METC 05/105) was approved by the VuMC- Medical Ethical Committee (VU Medical Center, The Netherlands; August, 2005). The study within the therapeutic foster care project (METC 09/046) was approved by the AMC - Medical Ethical Committee (Academic Medical Center Amsterdam, The Netherlands; April, 2009). Informed consent was obtained from all participating families. The order of assessment is different for the two samples, because it concerns data from two different studies, with different study protocols. Children in the regular foster care project were recruited from foster care agencies in the Netherlands. Foster parents were interviewed by telephone about symptoms of attachment disorder at the start of the study (time in current family; *M* = 35.40 months, *SD* = 18.38). Within three weeks after the interview, foster parents and teachers completed questionnaires assessing children’s internalizing and externalizing problems. Children in the treatment foster care project were recruited from an academic center for child and adolescent psychiatry, at the department of treatment foster care in the Netherlands (AMC-De Bascule, Amsterdam). The project started when children entered treatment foster care, often implying that children had just been transferred to a new foster family. Foster parents and teachers filled out questionnaires assessing internalizing and externalizing problems when children were in the family for at least 6 weeks. This study adhered to this time period, because a new foster setting is often accompanied by a temporary decrease or increase of problems. Foster parents were interviewed within the third month after the start of the study to assess symptoms of attachment disorder, assuming this is a plausible period for the development of an attachment relation between child and foster parent [18].

### Measures

#### Low quality of care

All children have been exposed to low quality if care (i.e. pathogenic care), as they all have been separated from the biological parent and placed in foster care. Additional experiences of low quality of care varied among children, depending on whether or not they had been exposed to child maltreatment and the type of child maltreatment they had been exposed to. Information about children’s exposure to maltreatment was obtained by questionnaires completed by child welfare caseworkers for children in regular foster care. Caseworkers were asked if child records reported occurrences of physical abuse, sexual abuse and neglect (0 = no, 1 = yes). Records from the child protective services were used to collect information about child maltreatment in the treatment foster care sample. Child maltreatment was indicated by exposure to physical abuse, sexual abuse and/or neglect, following the Maltreatment Classification System (MCS; [19]). We used a translated version of the MCS (Jonkman, Bolle, Harten-Hoogendam, Boer & Lindauer: *Checklist Kindermishandeling*, Unpublished manuscript, 2009). The average agreement between observers (Kappa) for the MCS was .72 or higher for the subscales. In this study two researchers independently from each other classified children’s records. In cases of disagreement, the most accurate classification was coded in consultation with a third researcher.

#### Attachment symptoms

The Disturbance of Attachment Interview (DAI; Smyke and Zeanah: *Disturbances of Attachment Interview.* Tulane University; Unpublished manuscript, 1999) was used to assess symptoms of Reactive Attachment Disorder (RAD), based on eight items; five items assessing symptoms of inhibited attachment disorder (1. *differentiates among adults*, 2. *seeks comfort preferentially from a preferred caregiver*, 3. *responds to comfort from caregivers when hurt, frightened or distressed*, 4. *responds reciprocally with familiar caregivers* and 5. *regulates emotions well with ample positive and developmentally expected levels of irritability and/or sadness*) and three items assessing symptoms of disinhibited attachment disorder (1. *clearly checks back with caregiver after venturing away, especially in unfamiliar settings*, 2. *exhibits reticence with unfamiliar adults*, 3. *not willing to go of readily with relative strangers*). Items were coded 0 if the symptom was definitely not present, 1 if there was some evidence for the symptom and 2 if the symptom was definitely present. This study adhered to a score of 2 (symptom definitely present) on one of the items of the subscales, to identify children with symptoms, based on the scale analysis performed by Oosterman and Schuengel [16] as well as the clinical ratings of disinhibited symptoms in the study of Rutter and his colleagues [8]. Item 4 has been found to insufficiently load on any of the DAI subscales, therefore it was excluded from this study [16]. The interraters reliability (Kappa) was estimated based on the degree of agreement between the two interviewers for all dichotomous items, *k* ranged from .88 to 1.00). Previous research has revealed acceptable validity and internal consistency [11,12].

#### Psychopathology

The *Child Behavior Checklist* for ages 1.5 to 5 (CBCL1.5-5; [20]) and 6 to 18 (CBCL 6–18; [21]) was used to asses children’s internalizing, externalizing and total problems. Foster parents rated the occurrence of 100 (CBCL 1.5-5) respectively 113 items (CBCL 6–18), on a three point scale (0 = *not at all true*, 1 = *somewhat true*, 2 = *very true*). In the present study internal consistency for the CBCL 1.5-5 broadband syndrome scales internalizing problems (α = .92), externalizing problems (α = .94) and total problems (α = .94) was satisfactory. Cronbach’s alpha (α) of the CBCL version 6–18 for the broadband syndrome scales internalizing problems, externalizing problems and total problems was respectively .83, .95 and .94. The *Teacher Report Form* for ages 1,5-5 and 6–18 assesses children’s school functioning and behavioral problems, based on 100 (TRF 1.5-5) respectively 113 (TRF 6–18) items rated at a three point scale (0 = *not at all true*, 1 = *somewhat true*, 2 = *very true*). Depending on the age of the child, day-care providers or teachers completed this questionnaire. The internal consistency was satisfactory for the TRF 1.5-5 broad band syndrome scales internalizing problems (α = .90), externalizing problems, (α = .96) and total problems (α = .97). Cronbach’s alpha (α) of the 6–18 version broadband syndrome was .91 for internalizing problems, .96 for externalizing problems and .97 for total problems.

### Statistical plan

Preliminary analyses were conducted to compare children with and without symptoms of inhibited, disinhibited and mixed symptoms of attachment disorder on the multivariate domain of placement characteristics, using MANOVA. Then series of independent t-tests were performed to compare children with symptoms of inhibited, disinhibited and mixed symptoms with the reference group of children without symptoms of attachment disorder on placement characteristics separately. Pre-placement information (age at out of home placement and time in foster care) was missing for 5 children (3 with and 2 without symptoms). Based available pre-placement information upon potential confounders were identified. When a variable was associated to both the dependent and the independent variable, covariate analyses were performed. Cohen’s *d* was used to indicate effect sizes of significant associations. Then, to examine the first hypothesis a chi-square test including al four categories was performed. Subsequently separate Fisher’s exact tests were used to test if symptoms of attachment disorder were associated with specific indicators of low Quality of care. Odds ratio (OR) indicated the strength of significant associations. MANOVA was conducted to compare four categories on the multivariate domain of psychopathology. Then to compare the inhibited, disinhibited and mixed symptoms with children without symptoms series of independent t-tests were conducted.

## Results

### Preliminary analyses

One-third (*N* = 42, 33.3%) of the children in the total group met criteria for inhibited and/or disinhibited attachment disorder, 18.1% of children in RFC and almost half (47.7%) of children in TFC (see Table 1). Percentages of children with inhibited (χ^2^ = 6.71, *p* = .01, OR = 6.62) and mixed symptoms (χ^2^ = 7.77, *p* = .01, OR = 7.35) were significantly higher in the TFC condition, compared to the RFC condition. In subsequent analyses, we accounted for this difference when foster care condition was also associated with the independent variable (covariate-effect analyses). For disinhibited symptoms results revealed a trend towards higher percentages in TFC, compared to RFC (χ^2^ = 3.23, *p* = .07). Two indicators of low quality of care were also reported more frequently in the TFC condition, physical (χ^2^ = 27.43, *p* = .00) and sexual abuse (χ^2^ = 12.36, *p* = .00). Multivariate statistics showed that parents report of internalizing and total problems and teacher’s report of externalizing and total problems was more severe in the TFC condition, *F* (1, 94) = 5.01-8.56, *p* = .03-.00. Because foster care condition was related to both attachment category and psychological problems, we performed covariate-analyses when examining the relation between attachment category and psychological problems.

**Table 1:** Number and percentages of children with and without symptoms of attachment disorder and foster care condition (n = 126)

	**Without**	**Inhibition**	**Disinhibition**	**Mixed symptoms**
Regular foster care	50 (82%)	2 (3%)	7 (12%)	2 (3%)
Treatment foster care	34 (52%)	9 (14%)	12 (19%)	10 (15%)

To test our hypotheses the total study sample was divided in four groups, including; [1] children without symptoms of attachment disorder (*N* = 84, 66.7%), [2] children with inhibited symptoms only (*N* = 11, 8.7%), [3] children with disinhibited symptoms only (*N* = 19, 15.1%) and [4] children with both inhibited and disinhibited symptoms (*N* = 12, 9.5%). Numbers and percentages of these four groups for the two types of foster care are presented in Table 1.

We then analyzed whether gender, age, time since out of home placement, age at out of home placement, number of placements and time in current placement differed between children without symptoms, with inhibited symptoms, with disinhibited symptoms and with mixed symptoms in our total sample. No differences were found between the four categories, regarding gender. Multivariate analyses showed a significant difference between the four categories and time since out of home placement, *F* (3,117) = 2.71, *p* = .048 and age at out of home placement *F* (3,117) = 2.75, *p* = .046. Subsequently, series of *t*-test were performed using the *without symptoms* group as reference category. Independent samples t-tests suggested that children with inhibited symptoms were significantly older when placed out of the home of origin, *t* (90) = −2.83, *p* = .006, for a shorter time (in months) in foster care at time of assessment, *t* (90) = 2.54, *p* = .013 and for a shorter time in the current foster family *t* (93) = 2.14, *p* = .035), compared to children without inhibited symptoms (see Table 2). Cohen’s *d* revealed large effect sizes, respectively *d* = −0.90, *d* = 0.82 and *d* = 0.69. No covariates were identified, as there were significant associations between these variables with both the dependent and independent variables. Therefore we performed no covariate-analyses.

**Table 2:** Means and standard deviations for placement characteristics for children with and without symptoms of attachment disorder

	**Without**	**Inhibition**	**Disinhibition**	**Mixed symptoms**
	**(n = 84)**	**(n = 11)**	**(n = 19)**	**(n = 12)**
	M(SD)	M(SD)	M(SD)	M(SD)
Time since out of home placement	37.06 (20.71)	20.65 (14.69)*	40.86 (21.92)	39.63 (14.08)
Age at out of home placement	21.21 (21.71)	40.64 (18.15)**	22.25 (22.12)	26.10 (19.89)
Number of placements	2.36 (1.44)	3.00 (1.18)	2.72 (1.90)	2.50 (1.51)
Time in current placement	23.71 (20.87)	9.56 (18.40)*	19.67 (20.50)	18.48 (17.28)

Note: *p < .05, **p < .01. Significance level univariate statistics without symptoms as reference category.

### Low quality of care in relation to symptoms of inhibited, disinhibited and mixed attachment

Multiple testing revealed no significant associations between the four symptoms of attachment categories with indicators of low quality of care. Performing separate analyses wherein the *without symptoms* group was the reference category indicated no significant associations between specific indicators of low Quality of care and inhibited or mixed symptoms (see Table 3). Exploratory post-hoc analyses for the separate categories revealed a significant association between disinhibited symptoms and previous exposure to physical abuse (*χ*^2^ = 5.58, *p* = .018, OR = 3.32). In the group of children that had been exposed to physical abuse, 31% showed exclusively disinhibited symptoms, compared to 12% in the not physically abused group.

**Table 3:** Experiences of low quality of care in children with and without symptoms of attachment

		**Without**	**Inhibition**	**Disinhibition**	**Mixed symptoms**
	***n *** **(%)**	***n***	***n***	***n***	***n***
Physical abuse	44 (35.5)	24	5	11*	4
Sexual abuse	22 (17.7)	12	3	4	3
Neglect	77 (62.1)	46	8	14	9

Note: *p < .05. Significance level chi-square without symptoms as reference category.

### Associated psychopathology of symptoms of inhibited, disinhibited and mixed attachment

Multivariate statistics showed that the four categories differed in severity in the overall domain of psychopathology reported by parents and teacher’s, *F* (18, 267) = 2.15, *p* = .005. According to parents, children with disinhibited symptoms showed more severe problems compared to children without symptoms (*F* [1, 101] = 3.97-8.80, *p* = .049-.004). Children with mixed symptoms showed more severe problems, compared to children without symptoms based on both parents (*F* [1, 94] = 25.51-37.25, *p* < .001) and teacher’s (*F* [1, 74] = 4.49-8.22, *p* = .038-.005) report (see Table 4). Cohen’s *d* revealed effect sizes ranging from 0.50-1.55. Additional analyses revealed that problems in children with mixed symptoms were also significantly more severe compared to children with disinhibited symptoms solely, *F* (12, 178) = 3.00, *p* = .001. When controlled for foster care condition covariate analyses revealed an overall effect on psychopathology, *F* (18, 264) = 2.01, *p* = .009. However, teacher’s report of internalizing, externalizing and total problems became non-significantly associated with attachment categories. Compared to the RFC and total sample, parent’s report of internalizing, externalizing and total problems in the TFC sample were not linked to inhibited or disinhibited symptoms.

**Table 4:** Means and standard deviations of psychopathology in children with and without symptoms of attachment disorder

	**Without (*n*** **= 65)**	**Inhibited (*n*** **= 8)**	**Disinhibited (*n*** **= 13)**	**Mixed (*n*** **= 10)**
	**M(SD)**	**M(SD) *p***	**M(SD) *p***	**M(SD) *p***
*Parents report*				
Internalizing	51.74 (11.98)	56.43 (10.09)	57.48 (13.66)*	76.84 (17.04)***
Externalizing	51.97 (13.88)	55.09 (19.13)	61.14 (13.75)**	73.24 (12.45)***
Total	51.37 (12.10)	54.50 (15.52)	60.35 (13.68)**	75.97 (12.81)***
*Teacher’s report*				
Internalizing	50.56 (9.59)	49.65 (8.76)	55.18 (13.69)	61.05 (16.97)**
Externalizing	56.72 (14.21)	52.83 (17.42)	61.29 (12.39)	66.83 (12.91)*
Total	51.63 (10.12)	49.95 (14.73)	56.36 (11.53)	60.60 (10.53)*

Note: *p < .05, **p < .01, ***p < .001. Significance level univariate statistics without symptoms as reference category.

## Discussion

Only a few studies investigated differences between inhibited and disinhibited symptoms of attachment disorder. Neither have they been investigated in maltreated foster children. This study was one of the first to examine whether differences between inhibited and disinhibited symptoms found in a unique sample of institutionalized children [4,8,22-25], can also be found in children exposed to other forms of pathogenic care. With regard to processes leading to inhibited and/or disinhibited symptoms, multivariate testing was unable to identify different forms of pathogenic care leading to either inhibited, disinhibited or mixed symptoms. For exploratory purposes, associations between different forms of pathogenic and attachment symptom groups were tested. Post-hoc analyses showed more physical abuse in children with disinhibited symptoms, consistent with the inclusion of harsh parenting in the DSM 5 criteria for disinhibited social engagement disorder [26]. Evidence supporting our hypothesis that inhibited symptoms were associated with specific forms of pathogenic care that are characterized by the absence of a preferred caregiver, was not found. Results support our idea that, like has been found in institutionalized children [4], inhibited and disinhibited have different recovery trajectories. Disinhibited symptoms were less likely to decrease after improved caregiving (placement in foster care). Whereas, the negative association of inhibited symptoms with length in foster care and time in current placement, suggests that inhibited symptoms disappear after improvement of caregiving conditions.

In accordance with our second hypothesis, results showed that externalizing problems accompany disinhibited symptoms, not inhibited symptoms. Interestingly, disinhibited symptoms also showed associations with internalizing problems, in contrast to the common notion that disinhibition typically goes together with externalizing behaviors [14]. The association with internalizing problems may be explained by the percentage of disinhibited symptoms in the TFC condition. Children in TFC have been reported with more psychopathology overall. Because symptoms of disinhibited symptoms are associated with more problems and are less responsive to improvement of caregiving conditions we support the idea of Smyke and colleagues [25] that symptoms of disinhibited require more intensive foster care.

Furthermore, data revealed a co-existence of inhibited and disinhibited symptoms. Although this comorbid pattern was not associated with specific indicators of low quality of care, it was associated with increased internalizing, externalizing and total problems as reported by foster parents in both the RFC and TFC condition.

The association we found between inhibited symptoms and age at out of home placement was unexpected, given the fact that this hasn’t been reported before and it lacks theoretical support. It may be explained by our finding that children with inhibited symptoms were also the children that had spend the shortest time in high quality care, as indicated by the time since they were placed out of home. Also, they were the children whose placement in the current foster family had occurred most recently. This assumption is supported by previous findings of others that reported decreasing inhibited symptoms in improved caregiving environments [25].

The potential existence of a mixed type of inhibited and disinhibited symptoms was strengthened by findings from the current study. Outcomes showed a substantial percentage of children with mixed symptoms. In this specific population, mixed symptoms seem less likely to decrease in improved caregiving conditions. Also, psychological problems tend to be more severe in children with mixed symptoms. Because the Disturbance of Attachment Interview is a screening instrument, further diagnostic research in this group is encouraged. Although the co-existence of inhibited and disinhibited symptoms was previously assigned to methodological issues [14], the present study urges on the importance of further inspection based on the persistence of symptoms and associated psychological problems.

### Limitations

Generalization of results should be with reservation, because of the relatively small sample size and sometimes singular cases that were analyzed. Also measures that were used, were not thorough: e.g. observational data could improve the study. This study is further limited in ways of data gathering and handling regarding low quality of care. First, different methods were used in the two samples. Whereas methods used in the TFC sample were found reliable, no reliability data was available for children in RFC. Second, although forms of abuse often co-existed with neglect, the relatively small sample size of this study hampered us to examine the contribution of co-existing forms of Low Quality of Care. Third, retrospective data gathering as well as inadequate documentation may have led to an underestimation of reports of maltreatment and abuse.

Due to these limitation results should be interpreted with reservation. This was a preliminary study examining the generalizability of previous findings in institutionalized children, supporting a split-up of inhibited and disinhibited symptoms. This study was a first step, the outcomes pointed out that differences between inhibited and disinhibited may be more visible when it concerns associated psychological problems and recovery, rather than processes (forms of pathogenic care) leading to either inhibited and disinhibited symptoms.

## Competing interests

The authors declare that they have no competing interests.

## Authors’ contributions

RJLL obtained funding for the study, in cooperation with CS, MO and FB. Recruitment of participants, data gathering and data analyses are coordinated by CSJ and EAB, under supervision of all other authors. CSJ wrote the manuscript, in cooperation with the other authors. All authors have read and approved the final manuscript.

## References

[B1] American Psychiatric Association Diagnostic and Statistical Manual of Mental Disorders (4th ed, Text Revision) 2000 Washington DC: American Psychiatric Association

[B2] Zeanah CH Keyes A Settles L Attachment relationship experiences and childhood psychopathology Ann N Y Acad Sci 2003 1008 22 30 14998869 10.1196/annals.1301.003

[B3] Zeanah CH Smyke AT Koga SF Carlson E Attachment in institutionalized and community children in Romania Child Dev 2005 76 1015 1028 16149999 10.1111/j.1467-8624.2005.00894.x

[B4] Gleason MM Fox NA Drury S Smyke A Egger HL Nelson CA III Gregas MC Zeanah CH Validity of evidence-derived criteria for reactive attachment disorder: indiscriminately social/disinhibited and emotionally withdrawn/inhibited types J Am Acad Child Adolesc Psychiatry 2011 50 216 231 21334562 10.1016/j.jaac.2010.12.012 PMC4127428

[B5] O’Connor TG Rutter M Attachment disorder behavior following early severe deprivation: extension and longitudinal follow-up. English and Romanian Adoptees Study Team J Am Acad Child Adolesc Psychiatry 2000 39 703 712 10846304 10.1097/00004583-200006000-00008

[B6] O’Connor TG Marvin RS Rutter M Olrick JT Britner PA Child–parent attachment following early institutional deprivation Dev Psychopathol 2003 15 19 38 12848433 10.1017/s0954579403000026

[B7] Lyons-Ruth K Bureau JF Riley CD Atlas-Corbett AF Socially indiscriminate attachment behavior in the Strange Situation: convergent and discriminant validity in relation to caregiving risk, later behavior problems, and attachment insecurity Dev Psychopathol 2009 21 355 372 19338688 10.1017/S0954579409000376 PMC2666941

[B8] Rutter M Colvert E Kreppner J Beckett C Castle J Groothues C Hawkins A O'Connor TG Stevens SE Sonuga-Barke EJ Early adolescent outcomes for institutionally-deprived and non-deprived adoptees. I: disinhibited attachment J Child Psychol Psychiatry 2007 48 17 30 17244267 10.1111/j.1469-7610.2006.01688.x

[B9] Pears KC Bruce J Fisher PA Kim HK Indiscriminate friendliness in maltreated foster children Child Maltreat 2010 15 64 75 19502477 10.1177/1077559509337891 PMC2810349

[B10] Chisholm K A three year follow-up of attachment and indiscriminate friendliness in children adopted from Romanian orphanages Child Dev 1998 69 1092 1106 9768488

[B11] Smyke A Dumitrescu A Zeanah C Attachment disturbances in young children. I: the continuum of caretaking casualty J Am Acad Child Adolesc Psychiatry 2002 41 972 982 12162633 10.1097/00004583-200208000-00016

[B12] Zeanah CH Scheeringa M Boris NW Hellers SS Smyke AT Trapani J Reactive attachment disorder in maltreated toddlers Child Abuse Neglect 2004 28 877 888 15350771 10.1016/j.chiabu.2004.01.010

[B13] Giltaij HP Sterkenburg PS Schuengel C Psychiatric diagnostic screening of social maladaptive behaviour in children with mild intellectual disability: differentiating disordered attachment and pervasive developmental disorder behaviour J Intellect Disabil Res 2013 57 doi:10.1111/jir.12079 10.1111/jir.12079 23906477

[B14] Zeanah CH Gleason MM Reactive Attachment Disorder: A Review for DSM-IV 2010 Washington, DC: American Psychiatric Association

[B15] Scheeringa M Research diagnostic criteria for infants and preschool children: the process and empirical support J Am Acad Child Adolesc Psychiatry 2003 42 1504 1512 14627886 10.1097/01.chi.0000091504.46853.0a

[B16] Oosterman M Schuengel C Autonomic reactivity of children to separation and reunion with foster parents J Am Acad Child Adolesc Psychiatry 2007 46 1196 1203 17712243 10.1097/chi.0b013e3180ca839f

[B17] Oosterman M Schuengel C Attachment in foster children associated with caregivers’ sensitivity and behavioral problems Infant Ment Health J 2008 29 609 623 10.1002/imhj.20198 28636248

[B18] Stovall KC Dozier M The development of attachment in new relationships: single subject analyses for 10 foster infants Dev Psychopathol 2000 12 133 156 10847621 10.1017/s0954579400002029

[B19] Barnett DMJT&CD Cicchetti D, Toth L Defining Child Maltreatment: The Interface Between Policy and Research Advances in Applied Developmental Psychology: Child Abuse, Child Development, and Social Policy 1993 Norwoord, NJ: Ablex 7 73 Ref Type: Generic

[B20] Achenbach TM Rescorla LA Manual for the ASEBA Preschool Forms & Profiles 2000 Burlington VT: University of Vermont, Research Center for Children, Youth & Families

[B21] Achenbach TM Manual for the Child Behavior Checklist/4-18 and 1991 Profile 1991 Burlington VT: University of Vermont, Department of Psychiatry

[B22] Rutter M Kreppner J Sonuga-Barke E Emanuel Miller Lecture: Attachment insecurity, disinhibited attachment, and attachment disorders: where do research findings leave the concepts? J Child Psychol Psychiatry 2009 50 529 543 19298474 10.1111/j.1469-7610.2009.02042.x

[B23] Zeanah CH Smyke AT Attachment disorders in family and social context Infant Ment Health J 2008 29 219 233 10.1002/imhj.20176 28636104

[B24] Boris NW Hinshaw-Fuselier SS Smyke AT Scheeringa MS Heller SS Zeanah CH Comparing criteria for attachment disorders: establishing reliability and validity in high-risk samples J Am Acad Child Adolesc Psychiatry 2004 43 568 577 15100563 10.1097/00004583-200405000-00010

[B25] Smyke AT Zeanah CH Gleason MM Drury SS Fox NA Nelson CA Guthrie D A randomized controlled trial comparing foster care and institutional care for children with signs of reactive attachment disorder Am J Psychiatry 2012 169 508 514 22764361 10.1176/appi.ajp.2011.11050748 PMC4158103

[B26] American Psychiatric Association Diagnostic and Statistical Manual of Mental Disorders 2013 5 Arlington, VA: American Psychiatric Publishing

